# A review on mycogenic metallic nanoparticles and their potential role as antioxidant, antibiofilm and quorum quenching agents

**DOI:** 10.1016/j.heliyon.2024.e29500

**Published:** 2024-04-16

**Authors:** Jorddy N. Cruz, Saima Muzammil, Asma Ashraf, Muhammad Umar Ijaz, Muhammad Hussnain Siddique, Rasti Abbas, Maimona Sadia, Saba, Sumreen Hayat, Rafael Rodrigues Lima

**Affiliations:** a Laboratory of Functional and Structural Biology, Institute of Biological Sciences, Federal University of Pará, Belém, 66075-110, PA, Brazil Brazil; b Institute of Microbiology, Government College University, Faisalabad, Pakistan; c Department of Zoology, Government College University, Faisalabad, Pakistan; d Department of Zoology, Wildlife and Fisheries, University of Agriculture, Faisalabad, Pakistan; e Department of Bioinformatics and Biotechnology, Government College University, Faisalabad, Pakistan; f Department of Microbiology and Molecular Genetics, The Women University Multan, Mattital Campus, Multan, Pakistan

**Keywords:** Antibiotic resistance, Biofilm, Mycogenic, Nanoparticles, Quorum sensing

## Abstract

The emergence of antimicrobial resistance among biofilm forming pathogens aimed to search for the efficient and novel alternative strategies. Metallic nanoparticles have drawn a considerable attention because of their significant applications in various fields. Numerous methods are developed for the generation of these nanoparticles however, mycogenic (fungal-mediated) synthesis is attractive due to high yields, easier handling, eco-friendly and being energy efficient when compared with conventional physico-chemical methods. Moreover, mycogenic synthesis provides fungal derived biomolecules that coat the nanoparticles thus improving their stability. The process of mycogenic synthesis can be extracellular or intracellular depending on the fungal genera used and various factors such as temperature, pH, biomass concentration and cultivation time may influence the synthesis process. This review focuses on the synthesis of metallic nanoparticles by using fungal mycelium, mechanism of synthesis, factors affecting the mycosynthesis and also describes their potential applications as antioxidants and antibiofilm agents. Moreover, the utilization of mycogenic nanoparticles as quorum quenching agent in hampering the bacterial cell-cell communication (quorum sensing) has also been discussed.

## 1 Introduction

With the advancement of nanotechnology to an encouraging stage, an expectation is there to create nano sized materials with safety, reliability and of eco-friendly nature. This widespread need of nano-sized materials prompts the nanotechnologists to establish a symbiotic relationship with other disciplines to equip the novel technology for the development of particular size and shape [1]. Nanoparticles (NPs) always exhibit unique physicochemical, biological and optical attributes as compared to bulk material because of their smaller size and higher surface area [2,3].

**Fig. 1:**
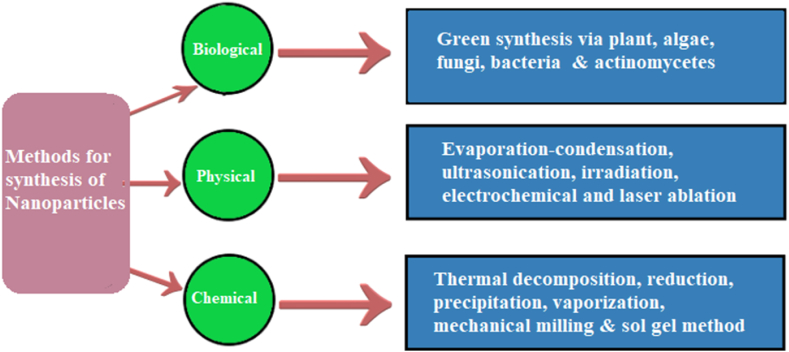
Numerous methods employed for the nanoparticle's synthesis including physical, biological and chemical means.

Various strategies such as biological, physical and chemical methods are established to synthesize transition and semiconductor metallic nanoparticles (Fig. 1). Among them, chemical methods were considered important because of their large-scale productions of nanoparticles [4]. However, at present, chemical methods employed to synthesize nanoparticles are outdated, costly, ineffective, utilize toxic materials, energy intensive and produced hazardous waste that poses harm to environment as well as ruled them out for any biomedical application. For example, the extensively employed method to generate silver nanoparticles was chemical based using reagents for the reduction of silver ions and to stabilize the nanoparticles. However, these reagents were quite toxic and might pose a harm to human health and environment [5], thus leading to growing interest in biogenic methods. On the other hand, in case of majority of the physical methods, a narrow size distribution of the particles or aggregates growing on a thin film was difficult to achieve. Furthermore, these methods were also time taking and not fully developed [6]. Therefore, synthesis of nanoparticles by green methods using biological resources such as fungi, plants, algae, bacteria and actinomycetes provides additional benefits over other methods, as they are reliable, cost effective, simple and eco-friendly, and can be easily used for the bulk production of nanoparticles [7]. Although various biological sources are available for the production of nanoparticles, but fungal mediated synthesis of nanoparticles represent marvellous scaffolds for this purpose because fungi release number of extracellular enzymes and their culturing is easy in laboratory [8].

On the other hand, the widespread increase in the occurrence of multidrug resistant (MDR) microbes and the continuous stress on the healthcare expenses requires to develop new and effective antimicrobial agents [9]. Secondly, most of the microbes have the potential to form biofilms, which makes it difficult to treat the microbial infections. Generally, biofilm is defined as a microbial consortium adhering to a surface and communicating with the other cells and gathering them to the surface through a self-producing matrix which is composed of extracellular polymeric substance (EPS). It has been reported that the microorganisms produce biofilms through the mechanism of quorum-sensing (QS). It is a cell-to-cell bacterial communication mechanism by which they monitor the density of their population by releasing and detecting signalling molecules called autoinducers (AIs). The microbial cells growing inside the biofilm matrix are persistent to many immunological events such as antibodies and phagocytes as well as towards the antibiotics thus culminating the host defense pathways [10]. Thus, development of effective antibiofilm and anti-QS strategies are required to overcome the challenges offered by biofilm formers. Some of the potential and currently used antibiofilm strategies involve disruption of mature biofilms, targeting by quorum quenching agents and combination of antibiotics with anti-QS compounds [10,11].

Fungal mediated metallic nanoparticles are potent in competing with the problems which arises due to these biofilm producers and MDR microorganisms. At present, mycogenic nanoparticles are focused on their antibiofilm and anti-quorum sensing activities due to their promising antimicrobial activities [11]. Nanoparticles exhibit antibiofilm potential by employing different mechanisms such as generation of reactive oxygen species (ROS), destruction of EPS and by inhibition of cellular communication (QS).

The present review aimed to provide an overview of fungal mediated synthesis of different nanoparticles, mechanism involved in their production and factors affecting their synthesis. Moreover, antioxidant, antibiofilm and anti-quorum sensing potential of these nanoparticles have also been discussed with the ultimate purpose of producing efficient antimicrobial agents.

## 2 Mycosynthesis of metallic nanoparticles

Nanoparticles synthesis using microorganisms have a promising potential and provides enormous advantages to mankind since they eliminate the utilization of toxic materials and are cost effective. One of the main advantages of metallic nanoparticles synthesis by green technology is their significant contribution to protect environment that is the ultimate goal of other green technologies [12,13]. Several microbes such as algae, bacteria, fungi and viruses are known for their potential to produce metal and metal oxide nanoparticles [14].

The mycosynthesis of metallic nanoparticles, also termed as myconanotechnology (MNT) involves fungi in nanotechnology to synthesize different nanoparticles. Fungi, due to their ease of maintenance, flexibility, tolerance, and efficiency as economic biological factories emerged as the most advantageous compared to other biosources for the production of nanoparticles [15]. Fungi belong to the kingdom of multicellular eukaryotes that are heterotrophs and are significant contributors in natural ecosystem. Fungi reproduction consists of both sexual and asexual phases, and may also have symbiotic relations with plant and bacteria [16]. It has been estimated that more than 6400 biologically active substances are synthesized by filamentous fungi such as ascomycetes, imperfect fungi and by other fungal genera [17]. They have been extensively utilized as stabilizing and reducing agents, since they exhibit heavy metal tolerance and do internalization and bioaccumulation of these metals. Moreover, cultivation of fungi on large scale (“nanofactories”) is also easy and they can produce nanoparticles having definite morphology and controlled size [[18], [19], [20]]. Other advantages include their ability to generate massive number of enzymes and proteins, that can be used for the rapid and sustainable production of nanoparticles [21,22]. Fungi contain various enzymes and proteins which act as reducing agents and thus can be explored for the mycosynthesis of metallic nanoparticles. Under same environmental conditions fungi usually grow faster when compared with bacterial cells. Undoubtedly, bacterial synthesis of metallic nanoparticles is common, but mycosynthesis would be more beneficial since fungal mycelia offer a huge surface area for interaction. Moreover, secretion of enzymes is much more in fungi in comparison to bacteria; thus, metal salts conversion into metallic nanoparticles is very speedy. Additionally, the fungal cell biopotential plays a significant role in the reduction and absorption of metal ions and in the ultimate generation of metallic nanoparticles [23].

## 3 Mechanism of mycosynthesis of metallic nanoparticles

**Fig. 2:**
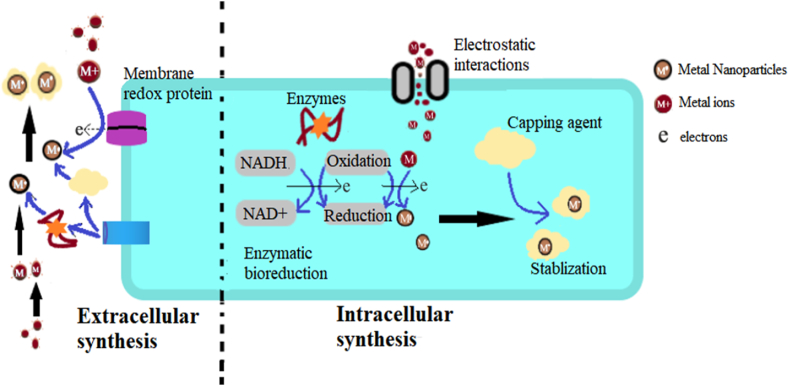
Methods for Mycosynthesis of metallic nanoparticles by releasing enzymes or metabolites inside (intracellular) or outside the fungal cell wall (extracellular).

The mechanism of biological synthesis of nanoparticles using fungi involves two distinct methods (such as extracellular and intracellular) that depends directly on the fungal metabolic nature to reduce metal ions (Fig. 2). During extracellular synthesis of metallic myconanoparticles, fungal cells release enzymes and-or metabolites outside the cell wall that will be utilized as a reducing agent in the reduction of metallic ion to fabricate myconanoparticles [9]. This is the most extensively used method, since no further processing is required to extract the nanoparticles from the cells [19,[24], [25], [26]] No doubt, the nanoparticles must be further purified to remove impurities and fungal residues that can be done by using simple methods such as gel filtration, membrane filtration, ultracentrifugation and dialysis [[27], [28], [29]]. On the other hand, during intracellular synthesis of myconanoparticles, the precursor (metal) is added to fungal mycelium and then internalized in the fungal biomass. After synthesis, nanoparticles are extracted through various methods such as filtration, centrifugation and chemical treatment that will disrupt the fungal cells and release the nanoparticles [30]. The whole procedure of intracellular synthesis of nanoparticles comprises of different steps. During the beginning stage of bioreduction, at the cell surface of fungi, metallic ions are trapped through electrostatic interaction of the positively charged ions of enzymes located on the fungal cell wall. While in the second stage, reduction of the metallic ions take place by the enzymes present in the cell wall, thus leading to the aggregation of metal ions and consequent generation of nanoparticles [31].

### 3.1 Mycosynthesis of nanoparticles by Fusarium

Several fungal species have been exploited successfully for the biosynthesis of nanoparticles (Table 1). However, the available literature clearly mentions that *Fusarium* species are the most widely used nanofactories. Numerous species of *Fusarium* like *Fusarium oxysporum*, *Fusarium acuminatum*, *Fusarium semitectum*, *Fusarium culmorum*, *Fusarium solani* etc. have been explored for the biosynthesis of metallic nanoparticles such as silver, platinum, gold, silica and palladium etc [[31], [32], [33], [34], [35]]. Different *Fusarium* sp. have been screened to select the potential species for the synthesis of metallic nanoparticles [36]. It was investigated that *F. oxysporum* generated silver nanoparticles (AgNPs) with very small diameter and *F. oxysporum* can reduce aqueous silver ions extracellularly to fabricate AgNPs [37,38]. Extracellular production of silver nanoparticles was also reported by other *Fusarium* and *Aspergillus* species. The production of nanoparticles was confirmed by UV–visible spectroscopy at maximum absorption spectra ranging between 420 and 450 nm, whereas SEM images demonstrated the spherical conformation of synthesized nanoparticles. These fungal based nanoparticles also hampered the growth of *E. coli*, *Staphylococcus* species and *Pseudomonas* species [39].

**Table 1:** Various fungal species involved in the fabrication of metallic nanoparticles.

Fungal sp.	Metallic NPs	Morphology & Synthesis conditions			Applications	References
		Size-Shape	Mode of Synthesis	Reaction Conditions		
*Fusarium acuminatum*	Ag	5–40 nm**,** Spherical	Extracellular	15 mL of 1 Mm AgNO_3_ + 1 mL of fungal mycelium, incubation at 28 ± 2 °C for 48–72 h	Antibacterial effects: *E. coli* (JM-103) (ATCC-394030, *S. typhi* ((MTCC-733), *S. aureus* (ATCC25923) *S. epidermidis* (ATCC-12228), by well diffusion method. zone of inhibtio obtained were 10 mm, 15 mm, 17 mm, 16 mm respectively.	[40]
*Fusarium oxysporum*	Au, MTPs	5–20 nm, face-centered cubic	Extracellular	Incubation in the dark for 5 days at 30 °C	Diethyl ether extract exhibited minimum to moderate activity against pathogens (Inhibition zone ranging 6.5–11.3 mm). Distilled water extracts displayed least activity contrary to *E. faecalis* and *E. coli.*	[41]
	Au	8–40 nm, Cubic	Extracellular	Incubation for a period of 72 h	/	[42]
	Ag	30 nm, Spherical	Extracellular	Reaction time of 72 h	/	[43]
	cd	5–15 nm, Spherical and occasionally triangular	Extracellular	Reaction with Ag^+^ ions for 72 h	/	[44]
	Zirconia	20–50 nm, Quasi-spherical	Extracellular	Calcination at 400 °C for 3 h	/	[45]
	Zirconia	3–11 nm, Nanocrystalline	Extracellular	Hydrolysis of the anionic complexes at room temperature	/	[32]
	Platinum	10–100 nm, Hexagons, circles, pentagons, and rectangles	Both extracellular and intracellular synthesis	Incubated at 4 °C for 25 min	/	[46]
	Ag	10–100 nm, Hexagons, circles, rectangles, pentagons, and squares	Extracellular	96 h of incubation of fungus at 25 °C, with shaking at120 rpm	/	[47]
	V_2_O_5_	10–20 nm, Spherical	Extracellular	25 °C shaken for 96 h under dark conditions at room temperature	V_2_O_5_NPs strongly inhibited both mycelial growth (20.3–67.3 %) and spore germination (64.8–89.9 %) dose-dependently. V_2_O_5_NPs showed strong cytotoxicity against breast cancer cell-line MCF-7 with an value of 55.89 *μ*g-mL. resulted in reduction of cancer cell.	[48]
*Fusarium solani*	Ag	16.23 nm, Spherical	Extracellular	Incubated at 28 °C and agitated at 120 rpm	/	[40]
*Penicillium* sp.	Ag	5–25 nm, Variable shape, mostly spherical	Extracellular	Incubation at 5 °C for 24 h	/	[49]
	Ag	25–30 nm, Spherical, well-dispersed	Extracellular	25 °C for 72h	Antibacterial activity against MDR *E. coli* and *S. aureus* displayed good results exhbiting maximum zone of inhibition of (17 mm and 16 mm) respectively.	[50]
	Ag	30 ± 5 nm, Spherical	Extracellular	Shaken for 96 h at room temperature under dark conditions	Silver-treated cotton fabric showed 99.01 % bacterial reduction of *S. aureus* and 99.26 % of *E.coli*. The antimicrobial activity of silver-treated cotton fabric was kept at over 98.77 % reduction level even after exposure to 20 consecutive home laundering conditions.	[51]
	MgO	7–40 nm, Spherical	Extracellular	Incubated for 5 days at 30 ± 2 °C and shaking at 150 rpm	Maximum deocolorization of textile effluent due to MgO treatments were 92.2 ± 0.3 %. Wastewater quality was increased due to treatments as reduction in values of COD, TDS, TSS, and conductivity.	[52]
*Penicillium brevicompactum*	Ag	23–105 nm, Spherical	Extracellular	Incubated in shaker at 180 rpm in dark conditions at 25 °C for 72h	/	[53]
	AgNO_3_	8–10 nm, Pyramidal, ellipsoidal and spherical	Extracellular	24 h of incubation with 1 mM AgNO3	Maximum antibaterial activity was observed at 5 mM for *S. aureus* (1 cm)*, E. coli* (1.4 cm) and *P. aeruginosa* (1.4 cm).	[54]
*Penicillium chrysogenum*	Au	5–100 nm, Spherical, triangle, and rod	Extracellular	shaker-incubation at 27–29 °C for 72 h	/	[55]
*Penicillium nalgiovense*	Ag	15.2 ± 2.6 nm, Spherical	Extracellular	Incubation at 25 °C with shaking (120 rpm) for 48 h.	/	[56]
*Aspergillus*	Lead	1.77–5.8 μm, Round	Extracellular	Incubation for period of 6 days	/	[57]
	Zn	50–120 nm, Spherical	Extracellular	96 h at 25 °C in orbital shaking incubator	/	[58]
*Aspergillus clavatus*	Ag	10–25 nm, Polydispersed spherical or hexagonal	Extracellular	Flask incubated on an orbital shaker at 200 rpm at room temperature	The results showed an average antibacterail activity against *P. fluorescens* and *E. coli*. of 5.83 μg ml(-1) and antifungal activity of 9.7 μg ml(-1) against *C. albicans.*	[1,30]
*Aspergillus flavus*	Ag	8.92–1.61 nm, Spherical	Extracellular	pH of 4.5 and spore concentration of 1.5 × 10^7^spore-L	/	[59]
*Aspergillus niger*	Ag	size ranging between hundreds on nanometers and micrometers, Spherical	Extracellular	Flask agitated at 25 °C in dark	Antibacterail activity for *S. aureus* was	[60]
					about 15 mm. Whereas for *B. subtilis, E. coli*, and *P. aeruginosa* were 11 mm, 10 mm and 14 mm. The wound healing activity of 10 % silver nanoparticle	
					ointment showed significant	
					wound contraction from 8th day onwards and achieved 100 % with wound closure after 13 days.	
*Aspergillus oryzae*	Iron	10–24.6 nm Spherical	Intracellular	Incubation in a dark room condition	Iron-enriched *A. oryzae* showed high relative bioavailability and lowered iron surges into the blood compared to FeSO_4_.	[61]
	Zn	Ranges between 10 and 100 nm, Spherical	Extracellular	Incubation at 25 °C in a dark room condition	Zinc nanofertilizer showed enhancement of enzymatic activity and fertilization. The grain yield at crop maturity was improved by 37.7 % due to application of zinc nanofertilizer.	[62]
	Se	55.0 nm, Sphere isotropic, poly-dispersed	extracellular	Incubation at 25 °C in a dark room condition	SeNPs showed antibacterial effects against *A. calcoaceticus (*15.0 mm) and S. aurus (16.6 mm) zone of inhibtion. Also sowed antifungal activity against *C. albicans* (15.3 mm) and *A. flavus* (29.6 mm) zones.	[63]
*A. nidulans*	Co	20.29 nm, Spherical	Extracellular	Incubated in dark	/	[64]
*Aspergillus clavatus*	Ag	550–650 nm, Irregular	Extracellular	Incubation at 25 °C in a dark room condition for 120 h	Antimicrobial effects and helpful in cancer treatment.	[65]
*Aspergillus niger*	Ag	3–30 nm, roughly spherical	Extracellular	Incubated for 72 h at 28 °C	The silver nanoparticles exhibited antibacterial activity against *Staphylococcus* and *Bacillus* sp. and *E. coli*, formed the zone of inhibition as 0.9, 0.8 and 0.8 cm. Antifungal activity against *A. niger* the zone of inhibition was 1.2 cm.	[66]
*Verticillium*	Ag	25 ± 12 nm, Spherical	Extracellular	Incubated for 72 h at 28 °C	/	[67]
	Au	20 nm, Spherical	extracellular	Incubated for 24–72 h at 28 °C	/	[31]
*Verticillium luteoalbum*	Au	100 nm, Hexagonal and triangular	Extracellular	Incubated for 24–72 h at 28 °C	/	[68]
*Verticilliun dahlia*	Au	40 nm, Polygonal	Extracellular	Rotary shaker with rpm of 120 for a period of 5–7 days.	/	[69]
Nigrospora oryzae	Au	6–18 nm, crystalline	Extracellular	Incubated at 24 °C for 8–9 days in dark	Antihelminthic activity was sowed alterations occurred in enzymes (AcPase, AlkPase, ATPase and 5′-Nu) of parasite due to gold nanoparticles.	[70]
*Saccharomyces cerevisiae*	Cadmium	2–3.6, uniform size	Extracellular	Incubated at 35 °C for 2 days	*In situ* bio-imaging in yeast cells suggests that biosynthesized QDs are biocompatible.	[71]
*Hormoconis resinae*	Au	3–20 nm, Spherical and nano-regime	Extracellular	Incubated at 26–35 °C for 5 days	/	[72]
*Coelomycetous phoma*	Ag	71·06 ± 3·48 nm -	Extracellular	Shaking Incubation	Ag-NPs showed antibacterial activity against *E. coli*, *P. aeruginosa* and *S. aureus* also enhanced antibacterial activity of antibiotics.	[73,74]
*Trichoderma asperellum*	Ag	13–18 nm, Spherical	Extracellular	Incubated in dark at 30 °C for 144 h	Silver nanoparticles showed reducing viral infectivity, probably by blocking interaction of virus with cell.	[75]
*Trichoderma reesei*	Ag	15–25 nm, Spherical	Extracellular	24 h, under continuous shaking at 150 rpm.	/	[76]
*Trichoderma atroviride*	Se	60.48 nm–123.16 nm, Lattice	Extracellular	Dark and static conditions for a period of seven days at 23 ± 2 °C	/	[77]
*T. asperellum,*	Cu	Size range of 10–100 nm, Spherical	Extracellular	Dark conditions for a period of seven days	Antimicrobial and anticancerous effects was showed by copper nanoparticles.	[65]
*Colletotrichum*	Au	20–40 nm, decahedral and icosahedral	Extracellular	Incubated at 30 °C for 24 h	/	[78]
*Colletotrichum gloeosporioides*	Ag	13–74 nm, Spherical(polydispersed)	Extracellular	Incubated at 30 °C	/	[79]
*Trichoderma* sp.	Ag	8–60 nm, Round and uniform	Extracellular	Incubated at 4 °C in dark room for 5 days	/	[80]
	Au	30 nm, Spherical	Extracellular	Incubation given periodically on 7th, 14th, and 21st day of incubation in dark at 25 °C	Antioxidant activity was observed but no relation was seen betwwen ROS and GSH levels.	[81]
	Zn	12–35 nm, Hexagons	Extracellular	Incubated at 28 ± 2 °C for 72 h	The isolates showed antibacterial activity against plant pathogenic bacteria; *Xanthomonas oryzae,* zone of inhibition ranged from (11 mm–25 mm). Also in co-culture	[82]
					secondary metabolites zone size increased upto several mm.	
*Volvariella volvacea*	Ag	15 nm, Spherical	Extracellular	Incubated at 25 °C for 25 h	/	[83]
	Au	20–150 nm, Spherical and hexagonal	Extracellular	Incubated at 25 °C for 6 h	/	[83]
*Phanerochaete chrysosporium*	Ag	Ranged between 10 and 100 nm, Varied shapes	Extracellular	Incubated for 24 h	The antibacterial activity of AgNPs can be controlled and improved, via adjusting sulfide concentration.	[84]
*Epicoccum nigrum, Amylomyces rouxii and Guignardia mangiferae*	Ag	Nanosized, Variable	Extracellular	26 ± 2 °C for 15–30 days	/	[85]

The bioproduction of AgNPs was also performed by using *F. oxysporum* [47] and the nanoparticles of variable sizes (10–100 nm) and shapes (hexagons, circles, pentagons, rectangles and squares) were generated by *F. oxysporum* both intracellularly and extracellularly. Ingle et al. [40] also reported the production of silver nanoparticles by pathogenic fungi *Fusarium solani.* It was observed that spherical shaped nanoparticles having average diameter of 16.23 nm were secured when fungal cell filtrate was added to silver nitrate solution.

In another study by Khalil et al. AgNPs were biofabricated from *P. chrysogenum* and *F. chlamydosporum* that also exhibited antifungal activities [86]. Since the exact molecular mechanism behind the generation of nanoparticles is not yet fully understood therefore, scientists are trying to explore the mechanism at molecular and cellular level. Mukherjee et al., described the generation of gold nanoparticles (AuNPs) extracellularly by *F. oxysporum* having diameter of 8–40 nm. These nanoparticles were produced by simply soaking the fungal biomass in aqueous AuCl^4−^ ions for a period of 72 h. The reduction of metal ion was confirmed by measuring absorbance at 545 nm using UV–visible spectroscopy [42]. Pourali et al. also reported the mycosynthesis AuNPs from *F. oxysporum* with great propensity of conjugation with β-Lactam antibiotics, thus making them a better detoxification agent [28]. *F. oxysporum* has number of advantages that makes it beneficial biosource for the synthesis of AuNPs such as their fast growth rate, safety, ease of processing and low-cost biomass management [87].

Zirconia nanoparticles were fabricated by using *F. oxysporum* as fungal biomass and regularly shaped spherical NPs having average diameter of 3–11 nm were obtained [32]. Similarly, zirconia NPs were generated by treating the fungus *F. oxysporum* mycelial extract with aqueous ZrF_6_^2-^ anions. The anionic complex hydrolysis at room temperature resulted in the generation of nanocrystalline zirconia [45,46].

Shabani et al. mentioned the successful vanadium oxide nanoparticles (V_2_O_5_NPs) production by green technology using *F. oxysporum* as fungal cell factory [48]. The synthesized NPs exhibited the maximum absorption peak at 410 nm. The mycogenic nanoparticles were spherical in shape with 10–20 nm size and have zeta potential of −35.09 mV, revealing that the capped biomolecules had highly negatively charged surface. The prepared nanoparticles also exhibited strong antifungal activities against numerous pathogenic fungi. Moreover, cytotoxicity assay revealed the potential toxicity against MCF-7 cancer cell-line as depicted by cell shrinkage, condensation of chromatin and DNA fragmentation.

The biomass of *F. oxysporum* can also be used for the synthesis of cadmium sulphate nanoparticles (CdSNPs) as reported by Ref. [44]. Extremely stable nanoparticles were produced extracellularly when fungal biomass was challenged to aqueous CdS solution. The synthesis of nanoparticles was confirmed by a colour change to bright yellow. The authors observed that the synthesis was due to reduction of sulphate ions by the fungal secreted enzymes and the extended stability of nanoparticles was dedicated to the occurrence of the proteins in solution which bound to the nanoparticle's surface and prevent their aggregation.

### 3.2 Mycosynthesis of nanoparticles by penicillium

*Penicillium* species have the potential to produce nanoparticles both extracellularly and intracellularly. Shaligram et al. worked on the in vitro biosynthesis of AgNPs through *P. fellutanum* isolated from coastal mangrove sediment by using AgNO_3_ as a substrate [53]. It was observed that nanoparticles produced by *Penicillium* had a negative zeta potential and were considerably stable at a pH > 8 due to the electrostatic repulsion [51]. *Penicillium nalgiovense* AJ15 strain was also used for the green synthesis of AgNPs as reported by Ref. [56]. In their study, the synthesis of AgNPs by the *P. nalgiovense* AJ15 cell free filtrate was done and cysteine containing proteins were major contributor in this non-enzymatic reduction of silver ions. Singh et al. also reported the synthesis of AgNPs by an endophytic *Penicillium* specie isolated from the healthy leaves of *Curcuma longa* [50]. In another study, Taha et al. reported the synthesis of AgNPs using *P. italicum*. The prepared nanoparticles also exhibited significant antioxidant activity and a dose-dependent antimicrobial potential against *E. coli*, *S. aureus* and *Candida albicans* [88].

*Penicillium* specie could also be effectively used for the synthesis of AuNPs. In a study, *Penicillium* sp. reduced and nucleated AuCl_4_^(−)^ ions, and resulted in intracellular synthesis of size-controlled AuNPs after exposure to HAuCl_4_ solution [49]. Another study reported the synthesis of gold nanoparticles by using fungal biomass of *P. chrysogenum* isolated from Ahar copper mine. Transmission electron microscopy (TEM) confirmed the intracellular formation of nanoparticles with the size range of 5–100 nm and having triangle, spherical and rod shapes [55].

Magnesium oxide nanoparticles (MgO NPs) can be biofabricated using *Penicillium* biomass. MgO NPs were produced from *P. chrysogenum* and used their insecticidal activity against malarial vector *Anopheles stephensi* [52].

### 3.3 Mycosynthesis of nanoparticles by aspergillus

Among the different fungal sources, *Aspergillus* is a very auspicious candidate for the fabrication of nanoparticles since more than 350 species of this genus exist having biological diversity and potential to secrete large amount of proteins. Nanoparticles of diverse shapes and sizes can be produced by *Aspergillus* species with unique physicochemical features such as increased solubility and thermostability, biocompatibility and stability over a wide range of pH.

Silver nanoparticles synthesis was demonstrated by Vigneshwaran et al. by incubating the cells of *Aspergillus flavus* with silver nitrate solution [84]. *Aspergillus niger* species isolated from soil were utilized to prepare AgNPs. In this study, the *A. niger* cell filtrate was challenged with silver nitrate solution and kept at 25 ^ͦ^C on a rotary shaker at 120 rpm. The synthesized nanoparticle's average diameter was 8.92 nm [89]. The extracellular mycosynthesis of AgNPs was also demonstrated by different workers using fungal biomass of *Aspergillus fumigatus, Aspergillus terreus, Aspergillus clavatus* [1,30,66,90]. An eco-friendly approach for the synthesis of Ag NPs using *Aspergillus tamarii* was also described by Ref. [61]. Their results demonstrated spherical shaped nanoparticles with diameter ranging from 25 to 50 nm as confirmed by scanning electron microscope (SEM).

Silver and gold nanoparticles employing *Aspergillus terreus* (soil and food mold) as biological source were also produced. FTIR analysis provided a detailed description of proteins that were capped and bound during the reaction. The synthesized nanoparticles also exhibited antibacterial properties as portrayed in SEM images where damage to bacterial membrane of *Staphylococcus* and *Bacillus* species could be observed [91].

*Aspergillus* species can also be explored for the synthesis of other metallic nanoparticles besides silver and gold. For example, the synthesis of magnesium, zinc, and titanium nanoparticles was reported by using six *Aspergillus* species belonging to *A. terreus*, *A. flavus*, *A. niger*, *Aspergillus tubingensis*, *Aspergillus oryzae* and *A. fumigatus* and adding precursor salts of nitrates, sulphates, oxides and chlorides [61,92]. Similarly, Hussain et al. [62] also investigated the synthesis of zinc oxide nanoparticles (ZnO NPs) using *A. oryzae*. They also described the immobilization of β-galactosidase from *A. oryzae* on ZnO NPs. Selenium nanoparticles were produced from aqueous extract of fermented lupin of *A. oryzae* by reducing the selenium ions into isotropic, spherical and polydispersed selenium nanoparticles under gamma irradiation (30 kGy) [63]. Endophytic fungi *A. nidulans* was also exploited for the synthesis of cobalt oxide nanoparticles via detoxification process. For reduction and synthesis, nitrate reductase enzyme and other peptides were found to be involved [64].

### 3.4 Mycosynthesis of nanoparticles by verticillium

*Verticillium* species are soil-borne plant pathogens that are also explored for their synthesis of metallic nanoparticles. For the intracellular synthesis of silver nanoparticles, the fungal biomass of *Verticillium* sp. was challenged to the solution of metal ion that reduced the metal and produced silver myconanoparticles intracellularly having average diameter of 25 nm. The metal ions reduction was attributed to the presence of enzymes with in the fungal cell wall and the proliferation of fungal cells also confirmed the non-toxic nature of silver metal ions [67]. Gold nanoparticles were prepared by the exposure of *Verticillium* sp. to aqueous solution of AuCl_4_ that resulted in the salt reduction to gold NPs having average diameter of 20 nm. The maximum absorption peak was observed at 540 nm that confirmed the synthesis of nanoparticles [31]. Another study mentioned the production of gold nanoparticles having average size of 40 nm using saprophytic fungi *Verticilliun dahlia* [69].

Intracellular production of silver nanoparticles was also carried out by screening a variety of yeast, bacteria and fungi. *Pichia jadinii* and *Verticillium luteoalbum* exhibited the most promising results. The results revealed the intracellular production and accumulation of AgNPs having diameter of around 25 nm as confirmed by transmission electron microscopy (TEM) [68].

### 3.5 *Mycosynthesis of nanoparticles by Trichoderma*

*Trichoderma* species are broadly present in the soil and have been used as plant growth promoters and biocontrol agents [93,94]. These species can also be used for the biofabrication of nanoparticles by using various enzymes such as reductases. For example, the extracellular enzyme NADH-dependent nitrate reductase secreted by *Trichoderma reesei* was used for the reduction of silver ions into AgNPs [76]. Besides reductases, other metabolites can also play key role during reduction by employing them as electron carriers. Vahabi and Dorcheh, in their study described the role of quinine, anthraquinones and naphthoquinones as electron carrier during the reduction of silver into metallic nanoparticles [22]. In *T. asperellum*, amino and aromatic groups of different secondary metabolites were also reported to act as reducing or encapsulating agents for the synthesis of copper oxide nanoparticles (CuO NPs) [65].

Various *Trichoderma* species such as *T. harzianum, T. longibrachiatum, T. asperellum, T. pseudokoningii* and *T. virens* were exploited for the production of AgNPs. These nanoparticles were found aggregated or single having uniform shape and diameter ranging from 8 to 60 nm [80]. The biosorption capacity of *Trichoderma* sp. was explored for the synthesis of gold nanoparticles (AuNPs) and the synthesized nanoparticles were found to have an average diameter of 30 nm [81].

Metallic nanoparticles synthesized by *Trichoderma* species display strong antimicrobial activities against various microbes such as *Aspergillus, Fusarium, Pseudomonas*, and *Xanthomonas* [[82], [95], [96], [97], [98]]. In another study, ZnO NPs were biofabricated using three different Trichoderma species and their antimicrobial efficacy was checked against *Xanthomonas oryza*e pv. Oryzae. The results revealed the fabrication of nanoparticles with unique shapes (peaks and hexagons) and diameter ranging from 12 to 35 nm [82].

Three fractions (cell wall debris, culture filterate and cell lysate) from *Trichoderma atroviride* were used for the mycosynthesis of selenium nanoparticles (SeNPs). The prepared nanoparticles exhibited excellent antifungal activities against *Pyricularia grisea* and decreased the infection of *Alternaria solani* and *Colletotrichum capsici* on tomato and chilli leaves when applied doses were 100 and 50 ppm respectively [77].

### 3.6 Mycosynthesis of nanoparticles by other fungi

Fungal endophytes or mycoendophytes can also be used as efficient nanofactories for the synthesis of metallic nanoparticles since they have numerous extracellular enzymes and secondary metabolites that can participate in the synthesis process. Mycoendophytes are different fungal species living intracellularly in plants by establishing a symbiotic association. For the synthesis of metallic nanoparticles, different endophytes were explored such as *Epicoccum nigrum*, *Amylomyces rouxii and Guignardia mangiferae*. The results revealed the generation of Ag NPs exhibiting stability, biocompatibility and antimicrobial activity against variety of fungal and bacterial pathogens [85]. Undoubtedly, the size and shape of biologically synthesized nanoparticles depend on the biological species involved. For example, *Colletotrichum* sp. were explored for the production of essentially spherical NPs under identical conditions [78]. Moreover, *Colletotrichum gloeosporioides* could effectively synthesize AgNPs extracellularly by releasing reducing agent and other metabolites outside the cell wall [79].

The white rot fungus, *Phanerochaete chrysosporium* was also challenged with aqueous silver nitrate solutions and resulted in the formation of stable AgNPs [84]. Another study used the *coelomycetous Phoma* strain for the extracellular synthesis of AgNPs having diameter ranging from 60 to 80 nm [73,74]. Philip et al. (2009), reported the extracellular synthesis of different metallic nanoparticles such as silver, gold and Au–Ag nanoparticles by using the extract of *Volvariella volvacea*, as reducing and capping agents [83]. AuNPs were also prepared by treating mycelia free filtrate of *Nigrospora oryzae* with gold chloride and the average diameter of mycosynthesized nanoparticles was found to be 6–18 nm diameter [70]. Similarly, *Hormoconis resinae* was also found to be an excellent source for the fabrication of AuNPs with increased stability [72].

## 4 Factors affecting mycosynthesis of metallic nanoparticles

No doubt, biofabrication of nanoparticles offer various advantages over other methods, however, different studies have shown that polydispersity of nanoparticles is one of the major problems observed during biological synthesis. Thus, it is desirable to obtain nanoparticles having uniform size and morphology that can be carried out by adjusting the parameters for synthesis and cultivation. Studies have also shown that changes in pH, temperature, culture medium, metal precursor concentration, and the amount of biomass also resulted in nanoparticles with variable physicochemical properties [33,73]. Mycosynthesis is also directly influenced by various conditions, like pH, temperature, incubation time, fungal species biomass concentration and nature of metal species and their colloidal interaction conditions, that also control the localization, size, shape and dispersity of the nanoparticles formed.

### 4.1 Effect of pH on mycosynthesis of nanoparticles

pH is an important parameter affecting the mycosynthesis of nanoparticles as observed by a change in the conformation of nitrate reductase enzyme by variation in the quantity of protons in the reaction media that significantly changed the size and morphology of nanoparticles [54]. Similarly, Gericke and Pinches [68] also discovered that the potential of mycosyntheis by *Verticillium luteoalbum* was not affected by pH, however, variations in pH levels profoundly influenced the morphology and shape of the synthesized nanoparticles. They discovered that the nanoparticles produced by *V. luteoalbum* at pH 3 were mostly round in shape and their diameter was less than 10 nm. Whereas, at pH 5, small number of nanoparticles were spherical in shape and a substantial number of nanoparticles exhibited morphologies such as triangular, hexagons, spheres and rods. At pH 9, most of the nanoparticles had ill-defined morphologies and a few were spherical. The successful synthesis of nanoparticles is also affected by the pH as observed in case of myosynthesis of AgNPs by *Guignardia magniferae*. It was observed that at pH 1 and 4, no change in colour of silver nitrate solution was observed, whereas, at pH 5 and 6, a slight change in the colour of was observed. The successful synthesis and stability of nanoparticles was observed at neutral pH [91]. Another study also reported the influence of pH on the mycosynthesis of lead selenide nanoparticles (PbSe NPs) by using Trichoderma fungal biomass. The optimum pH for the synthesis of PbSe NPs was 8 where cubic faced nanoparticles having diameter of 10–30 nm were obtained, whereas, at pH 5 and 9, no synthesis was observed [99].

### 4.2 Effect of temperature on mycosynthesis of nanoparticles

It has been observed that temperature has a significant impact on the development of microbes along with the accumulation of heavy metals from the surrounding environment. Moreover, various attributes of mycogenic nanoparticles can be affected by fluctuation in temperature such as time of synthesis, stability and shape [100]. During the size-controlled synthesis of AgNPs by *F. oxysporum,* when the temperature was raised to 50 °C, the smallest sized nanoparticles (30.24 nm) were obtained [101]. However [102], observed an increase in the diameter of AgNPs by increasing the temperature when fungal biomass of *A. fumigatus* was used. They observed agglomerates having diameter of 322.8 nm at 25 °C, whereas size was increased to 1073.45 nm at 50 °C. The rate of synthesis of nanoparticles is also greatly influenced by a change in temperature. During the synthesis of AgNPs, by using the filterate of *T. harzianum*, the synthesis rate was maximum when the temperature was raised to 40 °C [18]. The impact of temperature is also notable while selecting the mechanism of mycosynthesis of nanoparticles (extracellular-intracellular). For the extracellular synthesis of cadmium sulphide (CdS) nanoparticles by using white rot fungus *Phanerochaete chrysosporium*, the optimum temperature was found to be 37 °C, whereas, for intracellular synthesis of these nanoparticles, a lower temperature (26 °C) was selected [103]. Nanoparticles stability can be lost at very high or very low temperature. The effect of temperature fluctuation on the synthesis and stability of AuNPs was checked and the results demonstrated that optimum temperature for synthesis was 37 °C, whereas at the higher temperatures of 45 °C and 55 °C, the stability and synthesis was decreased probably due to the inactivation of enzymes [104]. [105] also noted that at 10 °C and 80 °C, AgNPs were not synthesized using the filtrate of Rhizopus stolonifera due to inactivation and denaturation of enzymes and other metabolites.

### 4.3 Effect of biomass size on mycosynthesis of nanoparticles

One of the most important elements in the natural production of nanoparticles is the biomass saturation. The metabolites released by fungus, which act as reducing and capping agents for the synthesis and stability of nanoparticles, are solely responsible for fungal-mediated nanoparticles creation. Thus, biomass size is a critical element that must be considered to improve the optimal myco nanoparticle production. According to Ref. [50] changes in the percentage of fungal biomass in the precursor solution can affect the pace of such interaction as well as the production of nanoparticles. Intercellular production of silver nanoparticles was achieved by inoculating 5–20 g of wet biomass with 5 g of commensal fungus Penicillium sp. in a metal ion solution of 1 mM. Since the existence of catalysts was adequate for the formation of silver nanoparticles, the largest and the fastest yields were recorded in 15 and 20 g of biomass feedstocks without any agglomeration [50].In the fungal-mediated silver nanoparticle production, the effect of altering the amount of *F. oxysporum* biomass in the process was also investigated. Using 5–20 g of wet biomass with a difference of 5 g of fungus *F. oxysporum*, the influence of fungal biomass concentration was examined. The ideal fungal biomass was discovered to be 20 g, which was not only accountable for the particle amalgamation but also for silver ion bioreduction [23]. The effect of fungal biomass on the mycosynthesis of silver nanoparticles was also investigated by using the filtrate of *A. fumigatus.* [102] investigated the utilization of 1, 4, 7 and 10 g of fungal biomass and the optimum was found to be 7 g since the nanoparticles produced were more, having better dispersion and smaller in their size. Rose [106] also examined that more fungal biomass also increased the amount of AgNPs produced having uniform size distribution when *Penicillium oxalicum* was used. The increased production of nanoparticles was attributed to the increased rate of extracellular secretion of nitrate reductase enzyme by fungal mycelium.

### 4.4 Effect of metal ion concentration and other growth parameters on mycosynthesis of nanoparticles

Some other significant parameters that influence nanoparticles production in fungal-mediated synthesis is metal - ligand density. The key contributing component for the creation of fungal-mediated nanoparticles is thought to be metal ion concentrations. The optimal concentration of metal ions for the improved synthesis of AgNPs utilizing the fungus *Sclerotinia sclerotiorum* was examined in research employing varying amount of silver ions ranging from 0.2 to 2 mM. The results revealed that metal ion concentration of 2 mM was optimum for the considerable silver nanoparticles formation by *S. sclerotiorum* [33]. Kumari and colleagues employed *Trichoderma viride* fungus supernatant to make AuNPs by using two distinct concentrations of metal precursors (250 and 500 mg-L). The findings suggested that concentration of 250 mg-L was optimal for the production of AuNPs with *T. viride*. It was observed that at the greatest concentration of gold salt, the cell filtrate concentration drops, resulting in an inadequate capping as well as stabilizing action [107]. High concentrations of metal ions also resulted in large sized nanoparticles with uneven morphology probably due to the competition between metal ions and functional groups of fungal filtrate and also pose significant toxicity [106]. Several studies suggested that a lower concentrations of metal ions improved the dispersion of nanoparticles and also reduced the size of obtained nanoparticles [5]. By using the filtrate of *Rhizopus stolonifer* [105], prepared silver nanoparticles with smaller size (2 nm), by using the metal precursor at concentration of 10 mM, however, at concentrations of 100 and 1 mM, the obtained sizes were 54 and 14 nm respectively.

Fungi are known to display variable response depending on the growth conditions and culture media. For the mycosynthesis of nanoparticles, a culture media having substrates specific for enzymes involved in the reduction process can enhance their secretion thus encouraging the process of metal ion reduction and subsequent nanoparticles formation [101]. For the synthesis of AgNPs, *Sclerotinia sclerotiorum* was grown in various broths, and the results demonstrated that improved production of AgNPs was achieved by using potato dextrose medium [33].

### 4.5 Effect of retention time on mycosynthesis of nanoparticles

[107] found that the reaction time affects the shape of AuNPs produced by fungal-mediated metal nanoparticle synthesis. The experiment involved the fungus *Trichoderma viride* to decrease the gold metal ions during time periods of 24, 48, and 72 h. After 24 h, all of the nanoparticles were spherical with a diameter of 7–24 nm; whereas, after 48 h, a mixed population of spheres, triangles, and prisms with larger sizes such as 7–120 nm was witnessed. After 72 h, particles formed were predominantly triangles but also prisms with a size range of 20–400 nm. These findings implied that the smaller size of nanoparticles after 24 h of incubation was related to the commencement of nucleation process, followed by an increase in size and varied forms after 48 and 72 h of incubation owing to the crystal development. The study found that spheres might be combined to create triangles during crystal development, with additional fusion yielding bigger nanoparticles [107].

## 5 Antioxidant Activities of Mycogenic Metallic Nanoparticles

**Fig. 3:**
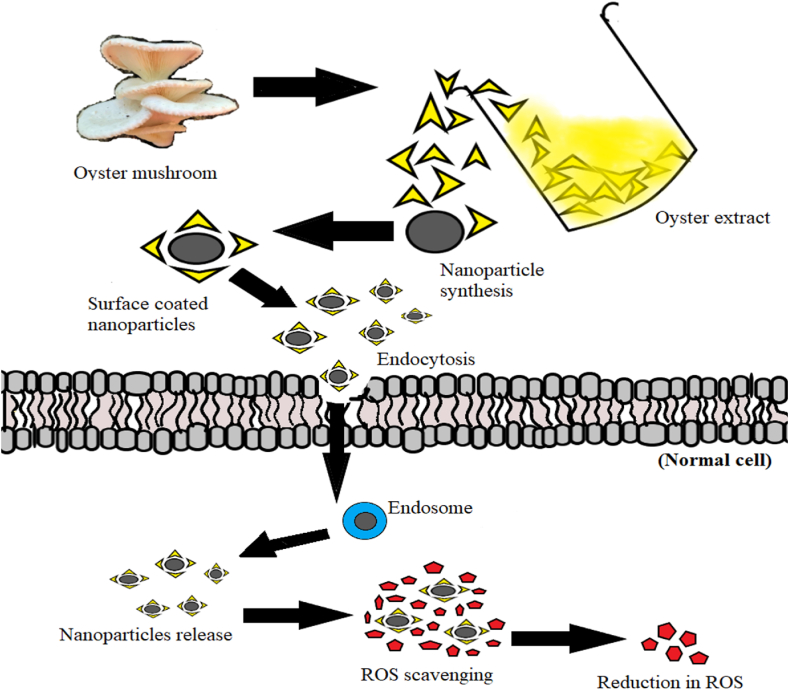
Antioxidant Activities of Mycogenic Metallic Nanoparticles. Nanoparticles get entry into the cell via endocytosis, and scavenge the ROS in order to reduce the damage caused by free radicals.

Excessive reactive free radicals have been generated in human body from various sources like mental stress, low diet, smoking, and other ailments. An antioxidant is a compound that strongly reduces or delays the oxidation mechanism whereas, the antioxidant activity measures the rate of inhibition of the oxidation process [108]. Antioxidants are substances such as nanoparticles preventing the production of free radicals as well as scavenging them while generated during different biochemical reactions in plants and animals thus playing a significant role in protecting against neurodegenerative and cardiovascular diseases and oxidative stress (Fig. 3). Determination of antioxidant potential will provide an insight regarding the interactions of target compound with biomolecules inside the living cell. Thus, it is useful to study the role of metal oxide nanoparticles as antioxidants because of their current applications and uses in biological systems [[109], [110], [111]].It has been observed that the antioxidant potential of biofabricated nanoparticles relies on the redox potential of flavonoid and phenolic compounds present on their surface [112,113].

Two mechanisms of the antioxidant activity have been proposed; (i) by the transfer of hydrogen atom, and (ii) single electron transfer by Ref. [114]. Excessive free radicals generated could be terminated or neutralized by donation of a hydrogen atom as in total oxyradical scavenging method, oxygen radical absorbance capacity, reduction of induced low-density lipoprotein oxidation and radical-trapping antioxidant parameters [115]. Contrary to this the single-electron transfer involves reducing the compounds like metals, radicals and carbonyls via transferring one electron. There is a change in colour as a result of reduction of the compound as observed in 2,2-diphenyl-1-picrylhydrazyl radical (DPPH) and Ferric Reducing Antioxidant Potential (FRAP) assays [114].

Metallic nanoparticles exhibited significant antioxidant potential in both extracellular and intracellular environments [116]. worked on the antioxidant potential of *P. pulmonarius* derived metabolite by evaluating their free radical scavenging ability through β-carotene-linoleate model and DPPH radical scavenging and assays. Their results demonstrated a dose-dependent radical scavenging activity and at a concentration of 2 mg-mL, 75 % inhibition of butylated hydroxyanisole (BHA) and 90 % inhibition of α-tocopherol was observed that was attributed to the presence of phenolic compounds in the extract*. P. eryngii* derived AgNPs were also tested for their free radical scavenging potential via DPPH, β-carotene-linoleate and ferrous ions reducing power assays and the results revealed that at a concentration of 10 mg-mL, the antioxidant capacities were 85 %, 77 % and 82 % respectively [117]. AgNPs biosynthesized by aqueous extract of *P. microspora* were also evaluated for their antioxidant potential and displayed a promising radical scavenging activity against H2O2 and 2,2′-diphenyl-1-picrylhydrazyl radicals with IC50 values of 94.95 ± 2.18 μg-mL, and 76.95 ± 2.96 respectively [118]. Biogenic AuNPs from *Cladosporium cladosporioides*, showed moderate antioxidant activity of 1.51 ± 0.03 mg of Ascorbic Acid Equivalent AAE-g sample [119].

The biofabricated AgNPs and AuNPs using fungal mycelia *of Pleurotus cystidiosis, Pleurotus citrinopileatus, Pleurotus ostreatus, Pleurotus eous, Pleuoruts flabellatus, Pleurotus eryngii, Pleurotus pulmonarius, Pleurotus florida* and *Schizophyllum commune* were tested for their antioxidant potential. The biofabricated AuNPs and AgNPs exhibited significant antioxidant potential and among the mycelial extracts used, *Pleurotus flabellatus* had the highest scavenging value of 415.93 mg in case of AuNPs whereas, the *Pleurotus flabellatus* extract had the highest radical scavenging value of 148.85 mg for AgNPs as shown in Fig. 3 [120]. In another study, *Cladosporium* species were used for the biosynthesis of AgNPs (CsAgNPs) and their antioxidant potential was investigated. CsAgNPs revealed the involvement of NADPH-dependent reductase in the generation of AgNPs and also displayed inhibitory activity against acetylcholine esterase (AChE) and butrylcholine esterase (BChE) [121].

A dose dependent antioxidant potential of chitosan nanoparticles (T-CSNPs) biofabricated by *Trichoderma harzianum* was observed. In addition to their antioxidant potential, biocompatibility and their bactericidal properties were also checked. It was observed that T-CSNPs were found soluble at a wide range of pH, displaying 100 % solubility (pH 1–3) and at pH 10, 72 % solubility was observed [65]. In another study, platinum nanoparticles fabricated with *F. oxysporum* were examined for their antioxidant, antimicrobial and photocatalytic activities. The antioxidant activity of platinum nanoparticles was found to be 79 % as measured by DPPH radical scavenging assay [122]. ZnO NPs synthesized by *Periconium* sp. extract also exhibited significant antioxidant property (85.52 % free radical quenching) at 100 μg-mL concentration [123]. Iron oxide nanoparticles synthesized from mycelium of *Penicillium* spp. were also checked for their antimicrobial and antioxidant potential. Various concentrations (0.625–160 μg-mL) of biofabricated iron oxide nanoparticles were used to measure their antioxidant potential by DPPH method and a potent DPPH scavenging potential was observed (63 %) as compared to standard ascorbic acid [124]. Another study reported the mycogenic synthesis of AgNPs from endophytic fungus *Talaromyces purpureogenus*, isolated from *Taxus baccata* Linn. The AgNPs displayed significant antibacterial potential against tested bacteria such as *Listeria monocytogenes, Escherichia coli, Shigella dysenteriae* and *Salmonella typhi.* These nanoparticles also displayed effective free radical scavenging activity against 2,2′-diphenyl-1-picrylhydrazyl. The promising antioxidant potential could be beneficial natural source of antioxidants that can balance the ROS and antioxidant levels to reduce the cell damage caused by oxidative stress [125].

## 6 Antibiofilm and anti quorum sensing activities of mycogenic metallic nanoparticles

Cytotoxic potential of metallic nanoparticles depends on their shape, size, capping-coating agents and the type of targeted pathogens. Biogenic nanoparticles generally display more toxicity as compared to nanoparticles synthesized by other routes. For the eradication of biofilm forming multidrug resistant bacteria and their planktonic counterpart, nanoparticles based biofilm inhibitors have been developed. The formation of biofilms acts as a primary step in pathogenesis for more than 80 % of bacterial infections [126]. Biofilm forming bacteria form a consortium mainly composed of proteins, polysaccharides, nucleic acids and-or hydrophobic substances such as lipopolysaccharides, lipids and surfactants that can attach to the surfaces and develop biofilm [127,128]. The biofilm formation enhances the survival chances of different bacterial species. The emerging increase in the antibiotic resistance underlines the significance of hunting the innovative antimicrobials. Various strategies can be employed by nanoparticles to exhibit antimicrobial activities against biofilm forming pathogens including oxidative stress induced by ROS, damage to cell wall due to electrostatic interaction, alteration of enzyme activity, membrane disruption and protein denaturation by the release of metal ions [129].

One of the current antimicrobial strategies involves the use of biologically derived nanomaterials as effective biofilm inhibitors. One of the studies demonstrated the role of AgNPs as biofilm inhibitors interacting with the biofilm compartments and penetrating the biofilm. Several factors can affect the antibiofilm activity of AgNPs such as surface chemistry, size distribution, hydrophobicity and surface charge [77].

On the other hand, quorum quenching (QQ) is a process that results in the intrusion of bacterial cross talk (QS) [130]. QS is responsible for various processes in bacteria such as virulence factors production, biofilm formation, sporulation, bioluminescence and bacteria-host interactions [131,132]. Metallic nanoparticles can act as potent anti QS inhibitors by affecting the mechanism of cell-cell communication. The strategies involving quorum quenching include the inhibition of signal molecules synthesis, inactivation or degradation of signalling molecules and interruption of signal molecule-receptor formation thus stopping the signal transduction cascade [133,134]. Although focusing on inhibition of biofilm formation and QS regulated virulence factors has been emerged as an effective and novel antimicrobial strategy yet there are limited studies that have reported the direct involvement of mycogenic metallic nanoparticles as QQ agents.

Several studies have demonstrated the potent anti QS potential of AgNPs thus inhibiting the formation of biofilm and other virulence factors. Various other types of biogenic metallic nanoparticles such as zinc, titanium, cerium, gold and silica nanoparticles also possess the significant antimicrobial potential and can effectively target QS cascade thus resulting in the biofilm inhibition [135].

**Fig. 4:**
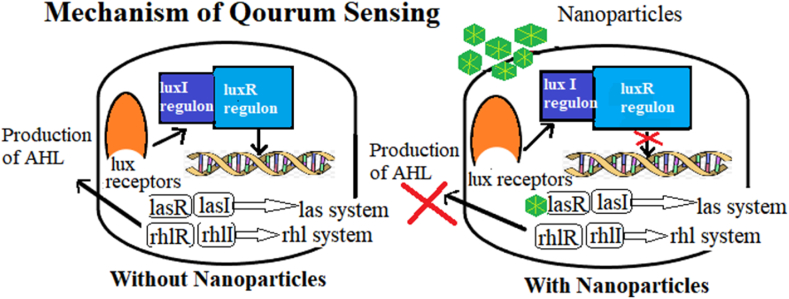
Mechanism of anti quorum sensing effect of mycogenic silver nanoparticles in *Pseudomonas aeruginosa* by inhibiting the expression of QS regulated genes.

AgNPs were fabricated by fungal metabolites of *Rhizopus arrhizus* BRS-07 and their quorum quenching activity was measured by targeting QS-regulated biofilm formation and virulence of *P*. *aeruginosa*. Disc diffusion antiquorum sensing assay by using *Chromobacterium violaceum* 12472 as bioindicator strain was performed in which the acylhomoserine lactone (AHL) regulated violet-coloured violacein production was checked by colorimetric method and the results revealed that violacein production was 100 % inhibited at a concentration of 25 μg-mL of mfAgNPs. Moreover, production of different virulence factors such as LasA protease, LasB elastase, siderophores, rhamanolipid, and pyocyanin was also reduced in a dose dependent manner. QQ potential of mycofabricated AgNPs was confirmed by the downregulation of the expression of genes regulated by QS pathway and the inhibition of expression of *lasI*, *lasR*, *lasA, lasB, rhlI*, *rhlR*, and *fabH2, phzA1* genes that is regulated by AHL-LasR complex as depicted in Fig. 4. The inhibitory effect of mycofabricated silver nanoparticles on the production of different virulence factors such as LasA protease, LasB elastase, rhamanolipid, and pyocyanin is also associated with *phzA1, rhlA and lasAB* operons [136].

AgNPs were also biofabricated using *A. flavus* (AfAgNPs) and *Emericella nidulans* (EnAgNPs) after reduction of AgNO_3_ by fungal mycelial extract and biofilm inhibition activity of fabricated nanoparticles was tested 96-wells microtiter plate method. The synthesized AgNPs significantly inhibited the biofilm formation by 80–90 % [137]. Biofilm inhibition was also observed by cerium oxide nanoparticles (CeO_2_) synthesized by phytopathogenic fungus *F. solani*. The inhibitory effect on biofilm formation by CeO_2_ nanoparticles was also examined by confocal laser scanning microscopy (CLSM). CLSM microscopy suggested that the inhibitory effects exhibited by the biofabricated CeO_2_ nanoparticles hinders the biofilm formation [138].

The intracellularly synthesized AuNPs from *Laccaria fraterna* mycelia displayed anti-quorum sensing potential against *Pseudomonas aeruginosa* and the inhibitory effect of AuNPs was observed on QS-regulated biofilm formation and the production of pyocyanin of *P*. *aeruginosa*. The quantitative and qualitative analysis revealed that AuNPs significantly hampered the pyocyanin production and biofilm formation [135].

Mycosilver nanoparticles were also generated by fungal extract of endophytic fungi *Setosphaeria rostrata*. Fourier transform infrared spectroscopy (FTIR) revealed the presence of alkaloids, phenols, flavonoids and proteins that were responsible for the capping and reduction processes. The results demonstrated that mycogenic silver nanoparticles significantly inhibited extracellular polysaccharide (EPS) and pyocyanin production, biofilms formation and swarming motility in *Pseudomonas aeruginosa* [139,140].

Furthermore, the antibiofilm potential of mycogenic AgNPs, that were produced by the enzymatic reduction of silver nitrate using the mycelial extract of endophytic fungus *Penicillium polonicum* ARA10 was also explored. The results revealed potent antibiofilm activity of mycogenic AgNPs on the biofilms of *A. baumannii*. The scanning electron microscopy (SEM) and field emission scanning electron microscopy (FE-SEM) images of biofilms embedded on the surface of central venous catheter samples confirmed that these AgNPs at minimum bactericidal concentration significantly destroyed the structure of biofilms and also resulted in the cell lysis [141]. *P. chrysogenum* fungal extracts were also explored for the synthesis of medically important copper oxide (CuO) and zinc oxide (ZnO) nanoparticles*.* These NPs showed significant antimicrobial potential not only against Gram positive bacteria and Gram negative bacteria but also against fungal phytopathogens. Quantitative and qualitative methods had displayed that these nanoparticles had effective antibiofilm potential against *S. aureus* [142].

Shobha et al. also reported the antibiofilm potential of ZnO NPs synthesized from *Trichoderma asperellum* isolated from rhizosphere soil. They demonstrated a significant increase in antiadherence and antibiofilm activity of nanoparticles by increasing their concentration [143].

The biofilm inhibitory potential of AgNPs prepared from *Aspergillus* sp. Silv2 extract and gold nanoparticles AuNPs fabricated from *Alternaria* sp. Gol2 extracts was also reported. These biogenic nanoparticles were further conjugated with chitosan to prepare chitosan–AgNPs and chitosan–AuNP conjugates. The antibiofilm potential of these conjugates was checked against four biofilm-forming bacteria such as *P. aeruginosa*, *E. coli*, *B. subtilis*, and *S. aureus*. The results displayed a significant antibiofilm potential of chitosan–AgNPs conjugate when compared with chitosan–AuNP conjugates [144].

The promising antibiofilm potential of biofabricated AgNPs from hyhal extract of *Alternaria* sp. against Gram positive and Gram negative bacteria was also reported [145]. These mycogenic nanoparticles were effective against *S. aureus*, *Salmonella abony, K. pneumoniae* and *Escherichia coli* having MIC50 values of 10.3, 22.69, 12.5 and 16.25 respectively. The biofilm inhibitory effect was also very promising and ranged between 16.66 and 64.81 %. In another study effect of mycogenic AgNPs generated from hyphal extract of *F. oxysporum* on growth, virulence factors production, expression of QS related genes and biofilm formation in *P. aeruginosa* strains PAO1 and PA14 was checked. The results displayed inhibitory effect on the expression of QS regulatory genes such as *lasR, lasI, rhlR, rhlI, pqsA* and *mvfR*. Moreover, there was a significant inhibition of elastase and rhamnolipids production, swarming and twitching motilities and of biofilm formation [140].

## 7 Conclusions and future perspectives

Fungal mediated synthesis of nanoparticles is found to be a suitable, efficient and eco-friendly approach with enormous potential and wide range of applications in different fields. Undoubtedly, fungal species used for the purpose of fabrication of nanomaterials should be generally safe. Interestingly, most of the fungal genera explored for the fabrication of important nanoparticles have generally recognized as safe (GRAS) status. These fungal species can be utilized for the synthesis of nanoparticles under different conditions of temperature, pH, biomass concentration, metal ions concentration etc. that resulted in nanoparticles having different physicochemical attributes. The mechanism of synthesis can be extracellular or intracellular depending on fungus and to broaden our knowledge regarding the mechanism of synthesis, research work on the generation of nanoparticles using purified enzymes and other fungal compounds should be carried out. A number of challenges are faced by the researchers in fungal nanobiotechnology that must be overcome to wider their applications for mankind. One of the major challenges in the mycofabrication of nanoparticles is that only few fungal genera have been explored for the fabrication purposes and a large number of genera are still unidentified. Mycogenic nanoparticles offer significant potential for their utilization as antioxidant, antibiofilm and anti QS agents in the control of antibiotic resistant pathogens. No doubt, inhibition of biofilm formation by targeting QS signalling cascade is a promising approach for the treatment of infections, however, further research is needed to understand the exact mechanism and for their future clinical applications. Moreover, in order to increase public acceptability of fungal mediated nanoparticles, more clinical research should be performed.

## Ethical approval

Ethical approval is not applicable to our study.

## Funding

R.R.L. is a researcher from 10.13039/501100003593Conselho Nacional de Desenvolvimento Científico e Tecnológico (CNPq) and received grant under number 312275/2021-8. The APC was funded by 10.13039/100017425Pró-Reitoria de Pesquisa e Pós-graduação from Federal University of Pará (PROPESP-UFPA).

## Data availability

No data was used for the research described in the article.

## CRediT authorship contribution statement

**Jorddy N. Cruz:** Writing – original draft, Data curation, Conceptualization. **Saima Muzammil:** Writing – original draft, Data curation, Conceptualization. **Asma Ashraf:** Writing – review & editing, Formal analysis. **Muhammad Umar Ijaz:** Writing – review & editing, Formal analysis. **Muhammad Hussnain Siddique:** Writing – review & editing, Formal analysis. **Rasti Abbas:** Formal analysis, Data curation. **Maimona Sadia:** Writing – review & editing, Software. **Saba:** Visualization, Formal analysis. **Sumreen Hayat:** Writing – review & editing, Supervision, Formal analysis, Conceptualization. **Rafael Rodrigues Lima:** Writing – review & editing, Validation.

## Declaration of competing interest

The authors declare that they have no known competing financial interests or personal relationships that could have appeared to influence the work reported in this paper.

## References

[1] Verma V.C., Kharwar R.N., Gange A.C. (2010). Biosynthesis of antimicrobial silver nanoparticles by the endophytic fungus *Aspergillus clavatus*. Nanomed.

[2] Pantidos N., Horsfall L.E. (2014). Biological synthesis of metallic nanoparticles by bacteria, fungi and plants. J. Nanomedicine Nanotechnol.

[3] Zinjarde S. (2012). Bio-inspired nanomaterials and their applications as antimicrobial agents. Chron. Young Sci..

[4] Murray C.B., Kagan C.R., Bawendi M.G. (2000). Synthesis and characterization of monodisperse nanocrystals and close-packed nanocrystal assemblies. Annu. Rev. Mater. Sci..

[5] Phanjom P., Ahmed G. (2017). Effect of different physicochemical conditions on the synthesis of silver nanoparticles using fungal cell filtrate of *Aspergillus oryzae* (MTCC No. 1846) and their antibacterial effect. Adv. Nat. Sci. Nanosci. Nanotechnol..

[6] Kaweeteerawat C., Ivask A., Liu R., Zhang H., Chang C.H., Low-Kam C., Fischer H., Ji Z., Pokhrel S., Cohen Y. (2015). Toxicity of metal oxide nanoparticles in *Escherichia coli* correlates with conduction band and hydration energies. Environ. Sci. Technol..

[7] Kumar V., Yadav S.K. (2009). Plant-mediated synthesis of silver and gold nanoparticles and their applications. J. Chem. Technol. Biotechnol. Int. Res. Process Environ. Clean Technol..

[8] Mishra A., Tripathy S.K., Wahab R., Jeong S.-H., Hwang I., Yang Y.-B., Kim Y.-S., Shin H.-S., Yun S.-I. (2011). Microbial synthesis of gold nanoparticles using the fungus *Penicillium brevicompactum* and their cytotoxic effects against mouse mayo blast cancer C 2 C 12 cells. Appl. Microbiol. Biotechnol..

[9] Fatima F., Verma S.R., Pathak N., Bajpai P. (2016). Extracellular mycosynthesis of silver nanoparticles and their microbicidal activity. J. Glob. Antimicrob. Resist..

[10] Packiavathy I.A.S.V., Priya S., Pandian S.K., Ravi A.V. (2014). Inhibition of biofilm development of uropathogens by curcumin–an anti-quorum sensing agent from *Curcuma longa*. Food Chem..

[11] Busi S., Paramanantham P. (2018). Metal and metal oxide mycogenic nanoparticles and their application as antimicrobial and antibiofilm agents. Fungal Nanobionics Princ. Appl..

[12] Omidi S., Sedaghat S., Tahvildari K., Derakhshi P., Motiee F. (2018). Biosynthesis of silver nanocomposite with Tarragon leaf extract and assessment of antibacterial activity. J. Nanostructure Chem..

[13] Ottoni C.A., Simões M.F., Fernandes S., Dos Santos J.G., Da Silva E.S., de Souza R.F.B., Maiorano A.E. (2017). Screening of filamentous fungi for antimicrobial silver nanoparticles synthesis. Amb. Express.

[14] Khandel P., Shahi S.K. (2018). Mycogenic nanoparticles and their bio-prospective applications: current status and future challenges. J. Nanostructure Chem..

[15] El Sayed M.T., El-Sayed A.S.A. (2020). Biocidal activity of metal nanoparticles synthesized by *Fusarium solani* against multidrug-resistant bacteria and mycotoxigenic fungi. J. Microbiol. Biotechnol..

[16] Duhan J.S., Kumar R., Kumar N., Kaur P., Nehra K., Duhan S. (2017). Nanotechnology: the new perspective in precision agriculture. Biotechnol. Rep..

[17] Berdy P. (2005). Bioactive microbial metabolites: a personal view. J. Antibiot. (Tokyo)..

[18] Ahluwalia V., Kumar J., Sisodia R., Shakil N.A., Walia S. (2014). Green synthesis of silver nanoparticles by *Trichoderma harzianum* and their bio-efficacy evaluation against *Staphylococcus aureus* and *Klebsiella pneumoniae*. Ind. Crops Prod. Complete.

[19] Azmath P., Baker S., Rakshith D., Satish S. (2016). Mycosynthesis of silver nanoparticles bearing antibacterial activity. Saudi Pharm. J..

[20] Rai M., Yadav A., Gade A. (2008). Current [corrected] trends in phytosynthesis of metal nanoparticles. Crit. Rev. Biotechnol..

[21] Alghuthaymi M.A., Almoammar H., Rai M., Said-Galiev E., Abd-Elsalam K.A. (2015). Myconanoparticles: synthesis and their role in phytopathogens management. Biotechnol. Biotechnol. Equip..

[22] Vahabi K., Dorcheh S.K. (2014). Biotechnol. Biol. Trichoderma.

[23] Khan A.U., Malik N., Khan M., Cho M.H., Khan M.M. (2018). Fungi-assisted silver nanoparticle synthesis and their applications. Bioprocess Biosyst. Eng..

[24] Gudikandula K., Vadapally P., Singara Charya M.A. (2017). Biogenic synthesis of silver nanoparticles from white rot fungi: their characterization and antibacterial studies. OpenNano.

[25] Sabri M.M., Möbus G. (2017). Electron beam transformation of glass nanoparticles. J. Phys. Conf. Ser..

[26] Silva L.P.C., Oliveira J.P., Keijok W.J., da Silva A.R., Aguiar A.R., Guimarães M.C.C., Ferraz C.M., Araújo J.V., Tobias F.L., Braga F.R. (2017). Extracellular biosynthesis of silver nanoparticles using the cell-free filtrate of nematophagous fungus *Duddingtonia flagrans*. Int. J. Nanomed..

[27] Ashrafi S.J., Rastegar M., Ashrafi M., Yazdian F., Pourrahim R., Suresh A. (2013). Influence of external factors on the production and morphology of biogenic silver nanocrystallites. J. Nanosci. Nanotechnol..

[28] Pourali P., Yahyaei B., Afsharnezhad S. (2018). Bio-synthesis of gold nanoparticles by *Fusarium oxysporum* and assessment of their conjugation possibility with two types of β-lactam antibiotics without any additional linkers. Microbiology.

[29] Qidwai A., Kumar R., Dikshit A. (2018). Green synthesis of silver nanoparticles by seed of *Phoenix sylvestris* L. and their role in the management of cosmetics embarrassment. Green Chem. Lett. Rev..

[30] Li X., Xu H., Chen Z.-S., Chen G. (2011). Biosynthesis of nanoparticles by microorganisms and their applications. J. Nanomater..

[31] Sastry M., Ahmad A., Khan M.I., Kumar R. (2003). Biosynthesis of metal nanoparticles using fungi and actinomycete. Curr. Sci..

[32] Bansal V., Rautaray D., Ahmad A., Sastry M. (2004). Biosynthesis of zirconia nanoparticles using the fungus *Fusarium oxysporum*. J. Mater. Chem..

[33] Nt K., J J. (2016). Optimization of reaction parameters for silver nanoparticles synthesis from *Fusarium oxysporum* and determination of silver nanoparticles concentration. J. Mater. Sci. Eng..

[34] Reyes L.R., Gómez I., Garza M.T. (2009). Biosynthesis of cadmium sulfide nanoparticles by the fungi Fusarium sp. Int. J. Green Nanotechnol. Biomed..

[35] Siddiqi K.S., Husen A. (2016). Fabrication of metal nanoparticles from fungi and metal salts: scope and application. Nanoscale Res. Lett..

[36] Dias M.A., Lacerda I.C.A., Pimentel P.F., de Castro H.F., Rosa C.A. (2002). Removal of heavy metals by an *Aspergillus terreus* strain immobilized in a polyurethane matrix. Lett. Appl. Microbiol..

[37] Ahmad A., Mukherjee P., Senapati S., Mandal D., Khan M.I., Kumar R., Sastry M. (2003). Extracellular biosynthesis of silver nanoparticles using the fungus *Fusarium oxysporum*. Colloids Surf. B Biointerfaces.

[38] Birla S.S., Gaikwad S.C., Gade A.K., Rai M.K. (2013). Rapid synthesis of silver nanoparticles from *Fusarium oxysporum* by optimizing physicocultural conditions. Sci. World J..

[39] Madakka M., Jayaraju N., Rajesh N. (2018). Mycosynthesis of silver nanoparticles and their characterization. MethodsX.

[40] Ingle A., Gade A., Pierrat S., Sonnichsen C., Rai M. (2008). Mycosynthesis of silver nanoparticles using the fungus *Fusarium acuminatum* and its activity against some human pathogenic bacteria. Curr. Nanosci..

[41] Deepa S., Kanimozhi K., Panneerselvam A. (2013). Antimicrobial activity of extracellularly synthesized silver nanoparticles from marine derived actinomycetes. Int J Curr Microbiol Appl Sci.

[42] Mukherjee P., Senapati S., Mandal D., Ahmad A., Khan M.I., Kumar R., Sastry M. (2002). Extracellular synthesis of gold nanoparticles by the fungus *Fusarium oxysporum*. Chembiochem Eur. J. Chem. Biol.

[43] Khosravi A., Shojaosadati S.A. (2009). Evaluation of silver nanoparticles produced by fungus Fusarium oxysporum. Int. J. Nanotechnol..

[44] Ahmad A., Mukherjee P., Mandal D., Senapati S., Khan M., Kumar R., Sastry M. (2002). Enzyme mediated extracellular synthesis of CdS nanoparticles by the fungus, *Fusarium oxysporum*. J. Am. Chem. Soc..

[45] Bharde A., Rautaray D., Bansal V., Ahmad A., Sarkar I., Yusuf S.M., Sanyal M., Sastry M. (2006). Extracellular biosynthesis of magnetite using fungi. Small Weinh. Bergstr. Ger..

[46] Riddin T.L., Gericke M., Whiteley C.G. (2006). Analysis of the inter- and extracellular formation of platinum nanoparticles by *Fusarium oxysporum* f. sp. lycopersici using response surface methodology. Nanotechnology.

[47] Mohammadian A. (2007). *Fusarium oxysporum* mediates photogeneration of silver nanoparticles. Sci. Iran..

[48] Gholami-Shabani M., Sotoodehnejadnematalahi F., Shams-Ghahfarokhi M., Eslamifar A., Razzaghi-Abyaneh M. (2021). Mycosynthesis and physicochemical characterization of vanadium oxide nanoparticles using the cell-free filtrate of *Fusarium oxysporum* and evaluation of their cytotoxic and antifungal activities. J. Nanomater..

[49] Kathiresan K., Manivannan S., Nabeel M.A., Dhivya B. (2009). Studies on silver nanoparticles synthesized by a marine fungus, *Penicillium fellutanum* isolated from coastal mangrove sediment. Colloids Surf. B Biointerfaces.

[50] Singh D., Rathod V., Ninganagouda S., Hiremath J., Singh A.K., Mathew J. (2014). Optimization and characterization of silver nanoparticle by endophytic fungi *Penicillium* sp. isolated from *Curcuma longa* (turmeric) and application studies against MDR *E. coli* and *S. aureus*. Bioinorg. Chem. Appl..

[51] Zhang F., Wu X., Chen Y., Lin H. (2009). Application of silver nanoparticles to cotton fabric as an antibacterial textile finish. Fibers Polym..

[52] Fouda A., Hassan S.E.-D., Abdel-Rahman M.A., Farag M.M.S., Shehal-deen A., Mohamed A.A., Alsharif S.M., Saied E., Moghanim S.A., Azab M.S. (2021). Catalytic degradation of wastewater from the textile and tannery industries by green synthesized hematite (α-Fe2O3) and magnesium oxide (MgO) nanoparticles. Curr. Res. Biotechnol..

[53] Shaligram N., Bule M., Bhambure R., Singhal R., Singh S., Szakacs G., Pandey A. (2013). Biosynthesis of silver nanoparticles using aqueous extract from the compacting producing fungi. Process Biochem..

[54] Nayak R.R., Pradhan N., Behera D., Pradhan K.M., Mishra S., Sukla L.B., Mishra B.K. (2011). Green synthesis of silver nanoparticle by *Penicillium purpurogenum* NPMF: the process and optimization. J. Nanoparticle Res..

[55] Sheikhloo Z., Salouti M., Katiraee F. (2011). Biological synthesis of gold nanoparticles by fungus epicoccumnigrum. J. Clust. Sci..

[56] Maliszewska I., Szewczyk K., Waszak K. (2009). Biological synthesis of silver nanoparticles. J. Phys. Conf. Ser..

[57] Pavani K.V., Kumar N.S., Sangameswaran B.B. (2012). Synthesis of lead nanoparticles by Aspergillus species. Pol. J. Microbiol..

[58] Kantabathini V.P. (2011). Biosynthesis of Zinc Nanoparticles by Aspergillus species. (n.d.).

[59] Ranjbar Navazi Z., Pazouki M., Halek F.S. (2010). Investigation of culture conditions for biosynthesis of silver nanoparticles using *Aspergillus fumigatus*, Iran. J. Biotechnol..

[60] Kalaiselvan V., Rajasekaran A. (2009). Biosynthesis of silver nanoparticles from *Aspergillus niger* and evaluation of its wound healing activity in experimental rat model. Int J Pharm Tech Res.

[61] Tarafdar J.C., Raliya R., Mahawar H., Rathore I. (2014). Development of zinc nanofertilizer to enhance crop production in pearl millet (pennisetum americanum). Agric. Res..

[62] Hussain A.I., Anwar F., Rasheed S., Nigam P.S., Janneh O., Sarker S.D. (2011). Composition, antioxidant and chemotherapeutic properties of the essential oils from two Origanum species growing in Pakistan. Rev. Bras. Farmacogn..

[63] Mosallam F.M., El-Sayyad G.S., Fathy R.M., El-Batal A.I. (2018). Biomolecules-mediated synthesis of selenium nanoparticles using *Aspergillus oryzae f*ermented Lupin extract and gamma radiation for hindering the growth of some multidrug-resistant bacteria and pathogenic fungi. Microb. Pathog..

[64] Vijayanandan A.S., Balakrishnan R.M. (2018). Biosynthesis of cobalt oxide nanoparticles using endophytic fungus *Aspergillus nidulans*. J. Environ. Manage..

[65] Saravanakumar A., Sadighi A., Ryu R., Akhlaghi F. (2019). Physicochemical properties, biotransformation, and transport pathways of established and newly approved medications: a systematic review of the top 200 most prescribed drugs vs. the FDA-approved drugs between 2005 and 2016. Clin. Pharmacokinet..

[66] Jaidev L.R., Narasimha G. (2010). Fungal mediated biosynthesis of silver nanoparticles, characterization and antimicrobial activity. Colloids Surf. B Biointerfaces.

[67] Mukherjee P., Ahmad A., Mandal D., Senapati S., Sainkar S.R., Khan M.I., Parishcha R., Ajaykumar P.V., Alam M., Kumar R., Sastry M. (2001). Fungus-mediated synthesis of silver nanoparticles and their immobilization in the mycelial matrix: a novel biological approach to nanoparticle synthesis. Nano Lett..

[68] Gericke M., Pinches A. (2006). Biological synthesis of metal nanoparticles. Hydrometallurgy.

[69] Iranmanesh S., Shahidi Bonjar G.H., Baghizadeh A. (2020). Study of the biosynthesis of gold nanoparticles by using several saprophytic fungi. SN Appl. Sci..

[70] Kar P.K., Murmu S., Saha S., Tandon V., Acharya K. (2014). Anthelmintic efficacy of gold nanoparticles derived from a phytopathogenic fungus. Nigrospora oryzae, PLoS ONE.

[71] Bao H., Hao N., Yang Y., Zhao D. (2010). Biosynthesis of biocompatible cadmium telluride quantum dots using yeast cells. Nano Res..

[72] Mishra A.N., Bhadauria S., Gaur M.S., Pasricha R. (2010). Extracellular microbial synthesis of gold nanoparticles using fungus Hormoconis resinae. JOM.

[73] Birla S.S., Tiwari V.V., Gade A.K., Ingle A.P., P Yadav A., Rai M.K. (2009). Fabrication of silver nanoparticles by *Phoma glomerata* and its combined effect against *Escherichia coli, Pseudomonas aeruginosa* and *Staphylococcus aureus*. Lett. Appl. Microbiol..

[74] Chen J.C., Lin Z.H., Ma X.X. (2003). Evidence of the production of silver nanoparticles via pretreatment of Phoma sp. 3.2883 with silver nitrate. Lett. Appl. Microbiol..

[75] Gaikwad S., Ingle A., Gade A., Rai M., Falanga A., Incoronato N., Russo L., Galdiero S., Galdiero M. (2013). Antiviral activity of mycosynthesized silver nanoparticles against herpes simplex virus and human parainfluenza virus type 3. Int. J. Nanomed..

[76] Gemishev O.T., Panayotova M.I., Mintcheva N.N., Djerahov L.P., Tyuliev G.T., Gicheva G.D. (2019). A green approach for silver nanoparticles preparation by cell-free extract from *Trichoderma reesei* fungi and their characterization. Mater. Res. Express.

[77] Joshi A.S., Singh V., Gahane A., Thakur A.K. (2019). Biodegradable nanoparticles containing mechanism based peptide inhibitors reduce polyglutamine aggregation in cell models and alleviate motor symptoms in a *Drosophila* model of huntington's disease. ACS Chem. Neurosci..

[78] Shankar S.S., Ahmad A., Pasricha R., Sastry M. (2003). Bioreduction of chloroaurate ions by geranium leaves and its endophytic fungus yields gold nanoparticles of different shapes. J. Mater. Chem..

[79] RavindraB K., Rajasab A.H. (2014). A comparative study on biosynthesis of silver nanoparticles using four different fungal species. https://www.semanticscholar.org/paper/A-comparative-study-on-biosynthesis-of-silver-using-Ravindra.B.Rajasab/d51cef830c61c91d9b096212f0072ef7e312aafc.

[80] Devi T.P., Kulanthaivel S., Kamil D., Borah J.L., Prabhakaran N., Srinivasa N. (2013). Biosynthesis of silver nanoparticles from Trichoderma species. Indian J. Exp. Biol..

[81] Nascimento T.S., Silva I.S.M., Alves M.C.M.A., Gouveia B.B., Barbosa L.M.R., Macedo T.J.S., Santos J.M.S., Monte A.P.O., Matos M.H.T., Padilha F.F., Lima-Verde I.B. (2019). Effect of red propolis extract isolated or encapsulated in nanoparticles on the in vitro culture of sheep preantral follicle: impacts on antrum formation, mitochondrial activity and glutathione levels. Reprod. Domest. Anim..

[82] Shobha B., Lakshmeesha T.R., Ansari M.A., Almatroudi A., Alzohairy M.A., Basavaraju S., Alurappa R., Niranjana S.R., Chowdappa S. (2020). Mycosynthesis of ZnO nanoparticles using Trichoderma spp. isolated from rhizosphere soils and its synergistic antibacterial effect against Xanthomonas oryzae pv. oryzae. J. Fungi.

[83] Philip D. (2009). Honey mediated green synthesis of gold nanoparticles. Spectrochim. Acta. A. Mol. Biomol. Spectrosc..

[84] Vigneshwaran N., Ashtaputre N.M., Varadarajan P.V., Nachane R.P., Paralikar K.M., Balasubramanya R.H. (2007). Biological synthesis of silver nanoparticles using the fungus *Aspergillus flavus*. Mater. Lett..

[85] Golinska P., Rathod D., Wypij M., Gupta I., Składanowski M., Paralikar P., Dahm H., Rai M. (2017). Mycoendophytes as efficient synthesizers of bionanoparticles: nanoantimicrobials, mechanism, and cytotoxicity. Crit. Rev. Biotechnol..

[86] Khalil N.M., Abd El-Ghany M.N., Rodríguez-Couto S. (2019). Antifungal and anti-mycotoxin efficacy of biogenic silver nanoparticles produced by *Fusarium chlamydosporum* and *Penicillium chrysogenum* at non-cytotoxic doses. Chemosphere.

[87] Naimi-Shamel N., Pourali P., Dolatabadi S. (2019). Green synthesis of gold nanoparticles using *Fusarium oxysporum* and antibacterial activity of its tetracycline conjugant. J. Mycol. Médicale..

[88] Taha Z.K., Hawar S.N., Sulaiman G.M. (2019). Extracellular biosynthesis of silver nanoparticles from *Penicillium italicum* and its antioxidant, antimicrobial and cytotoxicity activities. Biotechnol. Lett..

[89] Kumar P., Fennell P., Britter R. (2008). Measurements of particles in the 5-1000 nm range close to road level in an urban street canyon. Sci. Total Environ..

[90] Saravanan M., Nanda A. (2010). Extracellular synthesis of silver bionanoparticles from *Aspergillus clavatus* and its antimicrobial activity against MRSA and MRSE. Colloids Surf. B Biointerfaces.

[91] Balakumaran M.D., Ramachandran R., Balashanmugam P., Mukeshkumar D.J., Kalaichelvan P.T. (2016). Mycosynthesis of silver and gold nanoparticles: optimization, characterization and antimicrobial activity against human pathogens. Microbiol. Res..

[92] Klaus J., Kanton S., Kyrousi C., Ayo-Martin A.C., Di Giaimo R., Riesenberg S., O'Neill A.C., Camp J.G., Tocco C., Santel M., Rusha E., Drukker M., Schroeder M., Götz M., Robertson S.P., Treutlein B., Cappello S. (2019). Altered neuronal migratory trajectories in human cerebral organoids derived from individuals with neuronal heterotopia. Nat. Med..

[93] Contreras-Cornejo H.A., Macías-Rodríguez L., Cortés-Penagos C., López-Bucio J. (2009). Trichoderma virens, a plant beneficial fungus, enhances biomass production and promotes lateral root growth through an auxin-dependent mechanism in Arabidopsis. Plant Physiol.

[94] Hermosa R., Viterbo A., Chet I., Monte E. (2012). Plant-beneficial effects of Trichoderma and of its genes. Microbiol. Read. Engl..

[95] Bilesky-José N., Maruyama C., Germano-Costa T., Campos E., Carvalho L., Grillo R., Fraceto L., Lima R. (2021). Biogenic α-Fe 2 O 3 nanoparticles enhance the biological activity of Trichoderma against the plant pathogen *Sclerotinia sclerotiorum*. ACS Sustain. Chem. Eng..

[96] Consolo V.F., Torres-Nicolini A., Alvarez V.A. (2020). Mycosinthetized Ag, CuO and ZnO nanoparticles from a promising *Trichoderma harzianum* strain and their antifungal potential against important phytopathogens. Sci. Rep..

[97] Dutta P., Das G., Boruah S., Kumari A., Mahanta M., Yasin A., Sharma A., Deb L. (2021). Biol Forum Int J.

[98] Ponmurugan P. (2017). Biosynthesis of silver and gold nanoparticles using Trichoderma atroviride for the biological control of Phomopsis canker disease in tea plants. IET Nanobiotechnol..

[99] Diko C.S., Qu Y., Henglin Z., Li Z., Nahyoon N.A., Fan S. (2020). Biosynthesis and characterization of lead selenide semiconductor nanoparticles (PbSe NPs) and its antioxidant and photocatalytic activity. Arab. J. Chem..

[100] Elamawi R.M., Al-Harbi R.E., Hendi A.A. (2018). Biosynthesis and characterization of silver nanoparticles using *Trichoderma longibrachiatum* and their effect on phytopathogenic fungi, Egypt. J. Biol. Pest Control..

[101] Husseiny S.M., Salah T.A., Anter H.A. (2015). Biosynthesis of size controlled silver nanoparticles by *Fusarium oxysporum*, their antibacterial and antitumor activities. Beni-Suef Univ. J. Basic Appl. Sci..

[102] Shahzad A., Saeed H., Iqtedar M., Hussain S.Z., Kaleem A., Abdullah R., Sharif S., Naz S., Saleem F., Aihetasham A., Chaudhary A. (2019). Size-controlled production of silver nanoparticles by *Aspergillus fumigatus* BTCB10: likely antibacterial and cytotoxic effects. J. Nanomater..

[103] Borovaya M., Pirko Y., Krupodorova T., Naumenko A., Blume Y., Yemets A. (2015). Biosynthesis of cadmium sulphide quantum dots by using *Pleurotus ostreatus* (Jacq.) P. Kumm. Biotechnol. Biotechnol. Equip..

[104] Sreedharan S.M., Singh S.P., Singh R. (2019). Flower shaped gold nanoparticles: biogenic synthesis strategies and characterization. Indian J. Microbiol..

[105] AbdelRahim K., Mahmoud S.Y., Ali A.M., Almaary K.S., Mustafa A.E.-Z.M.A., Husseiny S.M. (2017). Extracellular biosynthesis of silver nanoparticles using *Rhizopus stolonifer*. Saudi J. Biol. Sci..

[106] Rose G.K., Soni R., Rishi P., Soni S.K. (2019). Optimization of the biological synthesis of silver nanoparticles using *Penicillium oxalicum* GRS-1 and their antimicrobial effects against common food-borne pathogens, Green Process. Synth. Met..

[107] Kumari S., Deori M., Elancheran R., Kotoky J., Devi R. (2016). In vitro and in vivo antioxidant, anti-hyperlipidemic properties and chemical characterization of *Centella asiatica* (L.) extract. Front. Pharmacol..

[108] Alam M.N., Bristi N.J., Rafiquzzaman M. (2013). Review on in vivo and in vitro methods evaluation of antioxidant activity. Saudi Pharm. J. SPJ Off. Publ. Saudi Pharm. Soc..

[109] Billotey C., Wilhelm C., Devaud M., Bacri J.c., Bittoun J., Gazeau F. (2003). Cell internalization of anionic maghemite nanoparticles: quantitative effect on magnetic resonance imaging. Magn. Reson. Med..

[110] Horak D., Babic M., Jendelová P., Herynek V., Trchová M., Pientka Z., Pollert E., Hájek M., Syková E. (2007). D-mannose-modified iron oxide nanoparticles for stem cell labeling. Bioconjug. Chem..

[111] Răcuciu M., Tecucianu A., Oancea S. (2022). Impact of magnetite nanoparticles coated with aspartic acid on the growth, antioxidant enzymes activity and chlorophyll content of maize. Antioxidants.

[112] Flieger J., Flieger W., Baj J., Maciejewski R. (2021). Antioxidants: classification, natural sources, activity/capacity measurements, and usefulness for the synthesis of nanoparticles. Materials.

[113] Mittal S., Khan M., Romero D., Wuest T. (2018). A critical review of smart manufacturing & industry 4.0 maturity models: implications for small and medium-sized enterprises (SMEs). J. Manuf. Syst..

[114] Wang L., Hu C., Shao L. (2017). The antimicrobial activity of nanoparticles: present situation and prospects for the future. Int. J. Nanomed..

[115] Kumar J., Kumar N., Sati N., Hota P.K. (2020). Antioxidant properties of ethenyl indole: DPPH assay and TDDFT studies. New J. Chem..

[116] Adebayo-Tayo B., Salaam A., Ajibade A. (2019). Green synthesis of silver nanoparticle using Oscillatoria sp. extract, its antibacterial, antibiofilm potential and cytotoxicity activity. Heliyon.

[117] Acay H., Yildirim A., Erdem Güzel E., Kaya N., Baran M.F. (2020). Evaluation and characterization of *Pleurotus eryngii* extract-loaded chitosan nanoparticles as antimicrobial agents against some human pathogens. Prep. Biochem. Biotechnol..

[118] Netala V.R., Kotakadi V.S., Bobbu P., Gaddam S.A., Tartte V. (2016). Endophytic fungal isolate mediated biosynthesis of silver nanoparticles and their free radical scavenging activity and anti microbial studies. 3 Biotech..

[119] Manjunath H.M., Joshi C.G., Raju N.G. (2017). Biofabrication of gold nanoparticles using marine endophytic fungus -. Penicillium citrinum, IET Nanobiotechnol.

[120] Madhanraj R., Eyini M., Balaji P. (2017). Antioxidant assay of gold and silver nanoparticles from edible basidiomycetes mushroom fungi. Free Radic. Antioxid..

[121] Popli D., Anil V., Subramanyam A.B., M N N., V R R., Rao S.N., Rai R.V., Govindappa M. (2018). Endophyte fungi, Cladosporium species-mediated synthesis of silver nanoparticles possessing in vitro antioxidant, anti-diabetic and anti-Alzheimer activity, Artif. Cells Nanomedicine Biotechnol..

[122] Gupta K., Chundawat T.S. (2019). Bio-inspired synthesis of platinum nanoparticles from fungus *Fusarium oxysporum*: its characteristics, potential antimicrobial, antioxidant and photocatalytic activities. Mater. Res. Express.

[123] Ganesan R.M., Gurumallesh Prabu H. (2019). Synthesis of gold nanoparticles using herbal Acorus calamus rhizome extract and coating on cotton fabric for antibacterial and UV blocking applications. Arab. J. Chem..

[124] Zakariya N.A., Jusof W.H.W., Majeed S. (2022). Green approach for iron oxide nanoparticles synthesis: application in antimicrobial and anticancer- an updated review. Karbala Int. J. Mod. Sci..

[125] Sharma A., Sagar A., Rana J., Rani R. (2022). Green synthesis of silver nanoparticles and its antibacterial activity using fungus *Talaromyces purpureogenus* isolated from *Taxus baccata* Linn. Micro Nano Syst. Lett..

[126] Beitelshees M., Hill A., Jones C.H., Pfeifer B.A. (2018). Phenotypic variation during biofilm formation: implications for anti-biofilm therapeutic design. Materials.

[127] Karygianni L., Ren Z., Koo H., Thurnheer T. (2020). Biofilm matrixome: extracellular components in structured microbial communities. Trends Microbiol..

[128] Kassinger S.J., van Hoek M.L. (2020). Biofilm architecture: an emerging synthetic biology target. Synth. Syst. Biotechnol..

[129] Baptista P.V., McCusker M.P., Carvalho A., Ferreira D.A., Mohan N.M., Martins M. (2018). Nano-strategies to fight multidrug resistant bacteria-"A battle of the titans". Front. Microbiol..

[130] Dong Y.-H., Gusti A.R., Zhang Q., Xu J.-L., Zhang L.-H. (2002). Identification of quorum-quenching N-acyl homoserine lactonases from Bacillus species. Appl. Environ. Microbiol..

[131] Davies D.G., Parsek M.R., Pearson J.P., Iglewski B.H., Costerton J.W., Greenberg E.P. (1998). The involvement of cell-to-cell signals in the development of a bacterial biofilm. Science.

[132] Li Y.-H., Tian X. (2012). Quorum sensing and bacterial social interactions in biofilms. Sensors.

[133] Defoirdt T., Pande G.S.J., Baruah K., Bossier P. (2013). The apparent quorum-sensing inhibitory activity of pyrogallol is a side effect of peroxide production. Antimicrob. Agents Chemother..

[134] LaSarre B., Federle M.J. (2013). Exploiting quorum sensing to confuse bacterial pathogens. Microbiol. Mol. Biol. Rev. MMBR..

[135] Samanta D., Guo H., Rubinstein R., Ramagopal U.A., Almo S.C. (2017). Structural, mutational and biophysical studies reveal a canonical mode of molecular recognition between immune receptor TIGIT and nectin-2. Mol. Immunol..

[136] Singh P., Kim Y.J., Singh H., Wang C., Hwang K.H., Farh M.E.-A., Yang D.C. (2015). Biosynthesis, characterization, and antimicrobial applications of silver nanoparticles. Int. J. Nanomed..

[137] Barapatre A., Aadil K.R., Jha H. (2016). Synergistic antibacterial and antibiofilm activity of silver nanoparticles biosynthesized by lignin-degrading fungus. Bioresour. Bioprocess..

[138] Kunga Sugumaran V., Gopinath K., Palani N., Arumugam A., Jose S., Sulthan A., Rajangam I. (2016). Plant pathogenic fungus *F. solani* mediated biosynthesis of Nanoceria: antibacterial and antibiofilm activity. RSC Adv..

[139] Akter M., Sikder MdT., Rahman MdM., Ullah A.K.M.A., Hossain K.F.B., Banik S., Hosokawa T., Saito T., Kurasaki M. (2018). A systematic review on silver nanoparticles-induced cytotoxicity: physicochemical properties and perspectives. J. Adv. Res..

[140] Saeki E.K., Martins H.M., de Camargo L.C., Anversa L., Tavares E.R., Yamada-Ogatta S.F., Lioni L.M.Y., Kobayashi R.K.T., Nakazato G. (2022). Effect of biogenic silver nanoparticles on the quorum-sensing system of *Pseudomonas aeruginosa* PAO1 and PA14. Microorganisms.

[141] Neethu S., Midhun S.J., Radhakrishnan E.K., Jyothis M. (2020). Surface functionalization of central venous catheter with mycofabricated silver nanoparticles and its antibiofilm activity on multidrug resistant Acinetobacter baumannii. Microb. Pathog..

[142] Mohamed R.M., Fawzy E.M., Shehab R.A., Abdel-Salam M.O., Salah El Din R.A., Abd El Fatah H.M. (2022). Production, characterization, and cytotoxicity effects of silver nanoparticles from Brown alga (*Cystoseira myrica*). J. Nanotechnol..

[143] Shobha, B B., Ashwini B.S., Ghazwani, M M., Hani U., Atwah B., Alhumaidi M.S., S M., Basavaraju S., Chowdappa S., Ravikiran T., Wahab S. (2022). Trichoderma-mediated ZnO nanoparticles and their antibiofilm and antibacterial activities. J. Fungi.

[144] Mostafa E.M., Abdelgawad M.A., Musa A., Alotaibi N.H., Elkomy M.H., Ghoneim M.M., Badawy M.S.E.M., Taha M.N., Hassan H.M., Hamed A.A. (2022). Chitosan silver and gold nanoparticle formation using endophytic fungi as powerful antimicrobial and anti-biofilm potentialities. Antibiotics.

[145] Baker A., Iram S., Syed A., Elgorban A.M., Bahkali A.H., Ahmad K., Sajid Khan M., Kim J. (2021). Fruit derived potentially bioactive bioengineered silver nanoparticles. Int. J. Nanomed..

